# Synthesis of silver nanoparticles and antibacterial property of silk fabrics treated by silver nanoparticles

**DOI:** 10.1186/1556-276X-9-216

**Published:** 2014-05-07

**Authors:** Guangyu Zhang, Yan Liu, Xiaoliang Gao, Yuyue Chen

**Affiliations:** 1 College of Textile and Clothing Engineering, Soochow University, Suzhou 215021, China; 2 Department of Textile Engineering, Yancheng Institute of Industry Technology, Yancheng 224005, China

**Keywords:** Silver nanoparticle, Multi-amino compound (RSD-NH_2_), Antibacterial activity, Silk fabric

## Abstract

A silver nanoparticle solution was prepared in one step by mixing AgNO_3_ and a multi-amino compound (RSD-NH_2_) solution under ambient condition. RSD-NH_2_ was in-house synthesized by methacrylate and polyethylene polyamine in methanol, which has abundant amino and imino groups. However, the characterization of silver nanoparticles indicated that these nanoparticles are easy to agglomerate in solution. Therefore, an *in situ* synthesis method of silver nanoparticles on the silk fabrics was developed. The examined results confirmed that the *in situ* synthesized silver nanoparticles were evenly distributed on the surface of fibers. The inhibition zone test and the antibacterial rate demonstrated that the finished fabrics have an excellent antibacterial property against *Staphylococcus aureus* and *Escherichia coli*. Moreover, the nanosilver-treated silk fabrics were laundered 0, 5, 10, 20, and 50 times and still retained the exceptional antibacterial property. When the treated fabrics were washed 50 times, the antibacterial rate is more than 97.43% for *S. aureus* and 99.86% for *E. coli*. The excellent laundering durability may be attributed to the tight binding between silver nanoparticles and silk fibers through the *in situ* synthesis. This method provides an economic method to enhance the antibacterial capability of silk fabrics with good resistance to washings.

## Background

With the development of science and technology and the improvement of the living standard, people have continuously strengthened their awareness on health and environmental protection of clothing [1]. Silk fabrics are highly popular with people for their excellent properties such as softness and gorgeous appearance, so they enjoy the honor as ‘The Queen of Fibers.’ However, silk fabrics provide an excellent environment for microorganisms to reproduce because of their large surface area and ability to retain moisture in the grids of fabrics. Therefore, to study and to improve the antibacterial properties of silk fabrics have an important influence on social significance and economic benefits [2-4].

To enhance the antibacterial properties of silk fabrics, silver nanoparticles are utilized to be attached onto the fabrics, although the mechanism is still in debate [5]. In previous studies, it was indicated that the use of a strong reductant such as borohydride promotes the formation of silver nanoparticles in the solution, which have a narrow size distribution. However, the severe deficiency confronted during the preparation of nanoparticles is the stability of the solution and the aggregation of nanoparticles [6-9]. In order to solve this problem, various methods are developed by researchers, such as the addition of surfactants (polyvinyl pyrrolidone and polyethylene glycol), spray pyrolysis, low plasma, and so on [5,10,11]. Nevertheless, the synthesis of a monodisperse and stable silver nanoparticle suspension is challenging and may go through tedious and complex procedures, which may hinder the practical applications of silver nanoparticles on textiles.

In this paper, we developed a method to synthesize a multi-amino compound (RSD-NH_2_) using methacrylate and polyethylene polyamine as a precursor with the presence of methanol [12].

**Figure 1:**
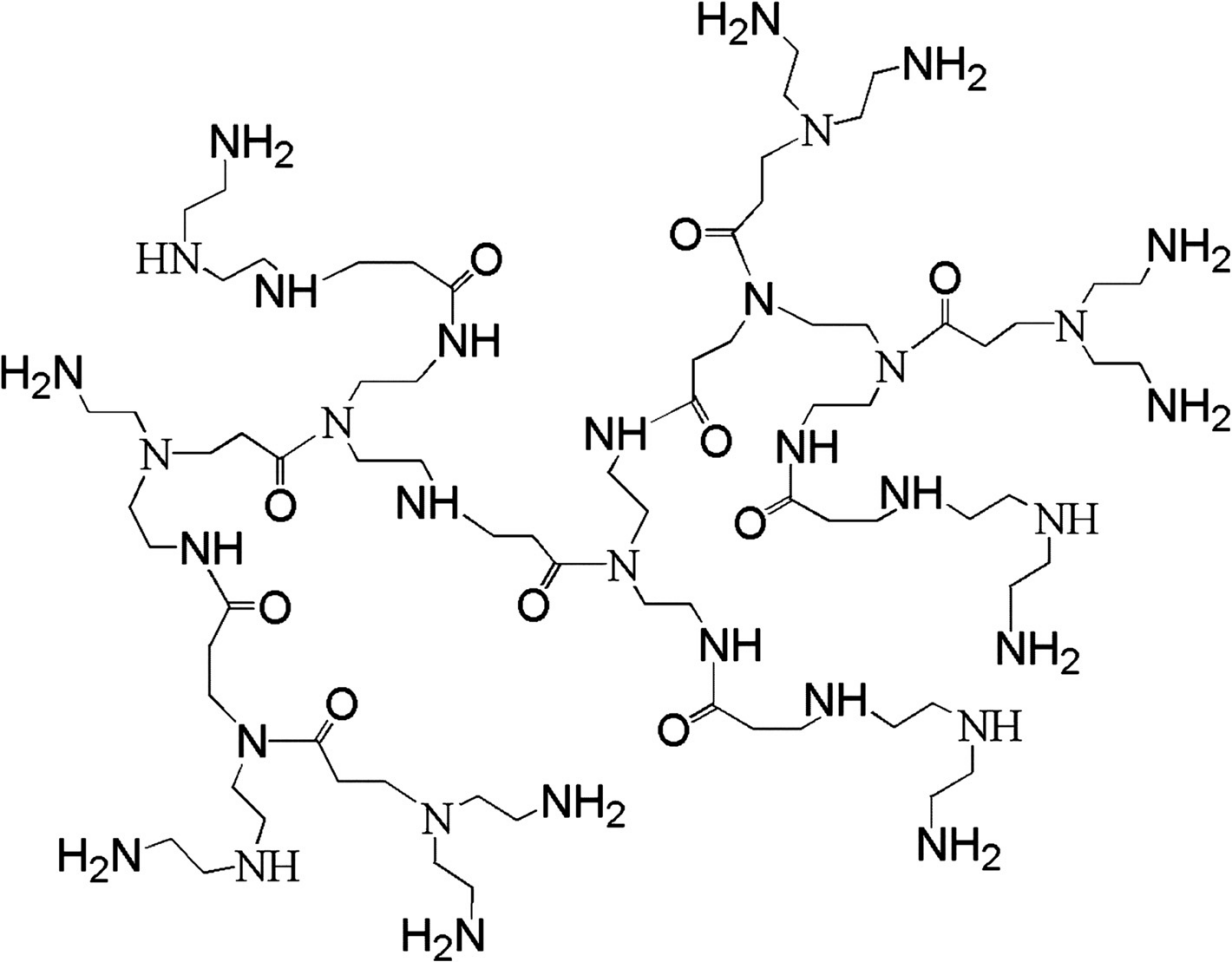
**Schematic description of the RSD-NH**_**2**_**'s molecular structure.**

The schematic description of the RSD-NH_2_'s molecular structure can be seen in Figure 1. We can see that a lot of amino and imino groups are on the surface of RSD-NH_2_, which can reduce silver ions to atoms and subsequently grow to silver nanoparticles [13]. The size distribution of particles and the properties of the solution are characterized. Furthermore, an *in situ* formation of silver nanoparticles on the silk fabrics is carried out to avoid the aggregation of particles in the solution [14]. The antibacterial property of silk fabrics was studied, particularly washed after different times.

## Methods

### Materials

The mass of mulberry silk fabric is 60 g/m^2^ (purchased from Xinchang Co. Ltd, Guangzhou, China). Methacrylate, polyethylene polyamine, methanol, sodium sulfide (Na_2_S), silver nitrate (AgNO_3_), and nitric acid (HNO_3_) in analytical grade were purchased from Sinopharm Chemical Reagent Co. Ltd. (Beijing, China). The multi-amino compound (RSD-NH_2_) was prepared in the laboratory. Nutrient broth and nutrient agar, which are both biochemical reagents to culture bacteria, were purchased from Scas Ecoscience Technology Inc. (Shanghai, China). *Staphylococcus aureus* (ATCC 6538) and *Escherichia coli* (ATCC 8099) were obtained from the College of Life Science, Soochow University (China).

### Synthesis of the multi-amino compound (RSD-NH_2_)

Polyethylene polyamine (1 M, 104 ml) was added in a 250-ml three-neck round-bottomed glass flask equipped with a constant-voltage dropping funnel, a thermometer, and a nitrogen inlet tube. The solution was stirred with a magnetic agitator. The flask was cooled to 24°C using a circulating water bath. Simultaneously, the mixture of methacrylate (1 M, 86 ml) in methanol was dropped slowly into the flask through the funnel. Afterwards, the reacting solution in the flask was removed from the water bath and left stirring for 4 h at room temperature. AB_2_-type monomers were synthesized, which made the solution present a light yellow color [15]. The solution was transferred to an eggplant-shaped flask and put into an automatic rotary vacuum evaporator. After evaporation of methanol under low pressure, the temperature was raised to 150°C using an oil bath to initiate the polymerization of the monomers. Eventually, a yellowish viscous multi-amino compound (RSD-NH_2_) was obtained with a 4-h polymerization.

### Preparation of the silver nanoparticles

Silver nitrate (AgNO_3_) and the multi-amino compound (RSD-NH_2_) were dissolved in deionized water, separately. Then AgNO_3_ aqueous solution was added dropwise into the RSD-NH_2_ solution under vigorous stirring. The initial concentrations of the reaction components were 0.017, 0.085, 0.17, and 0.255 g/l for AgNO_3_ and 2 g/l for RSD-NH_2_. The reacting mixture was kept stirring at room temperature until reduction of Ag^+^ to Ag was completed and brown silver nanoparticles appeared.

### Characterization of the silver nanoparticles

The size distribution and polydispersity of the silver nanoparticles were determined by dynamic light scattering (DLS) using a HPPS 5001 grain size analyzer (Malvern Instruments Ltd., Malvern, UK). Transmission electron microscopy (TEM) micrographs were obtained using a Tecnai G220 TEM (FEI Company, Hillsboro, OR, USA) operated at a 300-kV accelerating voltage. TEM samples were prepared by evaporating a drop of nanoparticle solution onto a 200-mesh copper grid, which was coated with a carbon support film. UV-visible (UV-vis) absorption spectra were recorded using an UV-3010 spectrophotometer (Shimadzu Ltd, Japan). K/S absorption spectra of treated silk fabrics were tested under a D65 illuminant at 10° observer using an Ultrascan XE spectrophotometer (HunterLab Co. Ltd., Reston, VA, USA). The X-ray diffraction (XRD) patterns of the silver nanoparticles were taken in the 2*θ* range of 20° to 80° at a scanning rate of 2°/min using Cu Kα radiation with a model D/max3c X-ray detector diffraction system (Rigaku Ltd, Japan).

For Fourier transform infrared (FTIR) analysis, the colloidal silver solution was poured into acetone and the resulting precipitates were dried for characterization. FTIR spectra were performed on a Nicolet 5700 FTIR spectrophotometer (Thermo Electron Corporation, USA).

### Preparation of silver nanoparticle-treated silk fabrics

The silk fabrics were immersed into the solution of mixed AgNO_3_ and RSD-NH_2_ at their respective concentration with the process of dipping and rolling twice. Subsequently, the fabrics were steamed for 30 min in a steam engine (BTZS10A, China). After that, the fabrics were washed by deionized water and dried at ambient temperature to produce the finished silk fabric.

### Antibacterial effect of nanoparticle-treated silk fabrics

The morphology and distribution of silver nanoparticles on the surface of fabrics were observed using a scanning electron microscope (SEM; S-570, Japan). The antibacterial effect of silver nanoparticle-treated silk fabrics was tested against *E. coli* and *S. aureus* by using a shaking flask method according to the antibacterial standard of knitted products (FZ/T 73023-2006, China). This standard specified the requirements of the antibacterial fabric, test methods, and inspection rules, which are applicable to the antibacterial fabrics made by natural fiber, chemical fiber, and blended fiber.

A sample fabric with a weight of 0.75 g was cut into small pieces with a size around 0.5 × 0.5 cm^2^ and was immersed into a flask containing 70 ml of 0.3 mM PBS (monopotassium phosphate, pH ≈ 7.2) culturing solution with a bacterium concentration of 1 × 10^5^ to 4 × 10^5^ colony-forming units (CFU)/ml. The flask was then shaken at 150 rpm on a rotary shaker at 24°C for 18 h. From each incubated sample, 1 ml of solution was taken and diluted to 10, 100, and 1,000 ml and then distributed onto an agar plate. All plates were incubated at 37°C for 24 h, and the colonies formed were counted by eyes. The percentage reduction was determined as follows (FZ/T 73023-2006, China):

$$\text{Reduction}\;\text{in}\;\text{CFU}\;\left(\%\right)=\frac{A-B}{A}\times 100\%,$$

where *A* and *B* are the bacterial colonies of the original silk fabrics and the silver-treated silk fabrics, respectively.

To evaluate the durability of the nanoparticle-treated silk fabrics against repeated launderings, AATCC Test Method 61-1996 was applied. An AATCC standard wash machine (Atlas Launder-Ometer) and detergent (AATCC Standard Detergent WOB) were used. Samples were cut into several 5 × 15 cm^2^ swatches and put into a stainless steel container with 150 ml of 0.15% (*w*/*v*) WOB detergent solution and 50 steel balls (0.25 in. in diameter) at 49°C for various washing times to simulate 5, 10, 20, and 50 wash cycles of home/commercial launderings.

## Results and discussion

### Synthesis of silver nanoparticles in solution

**Figure 2:**
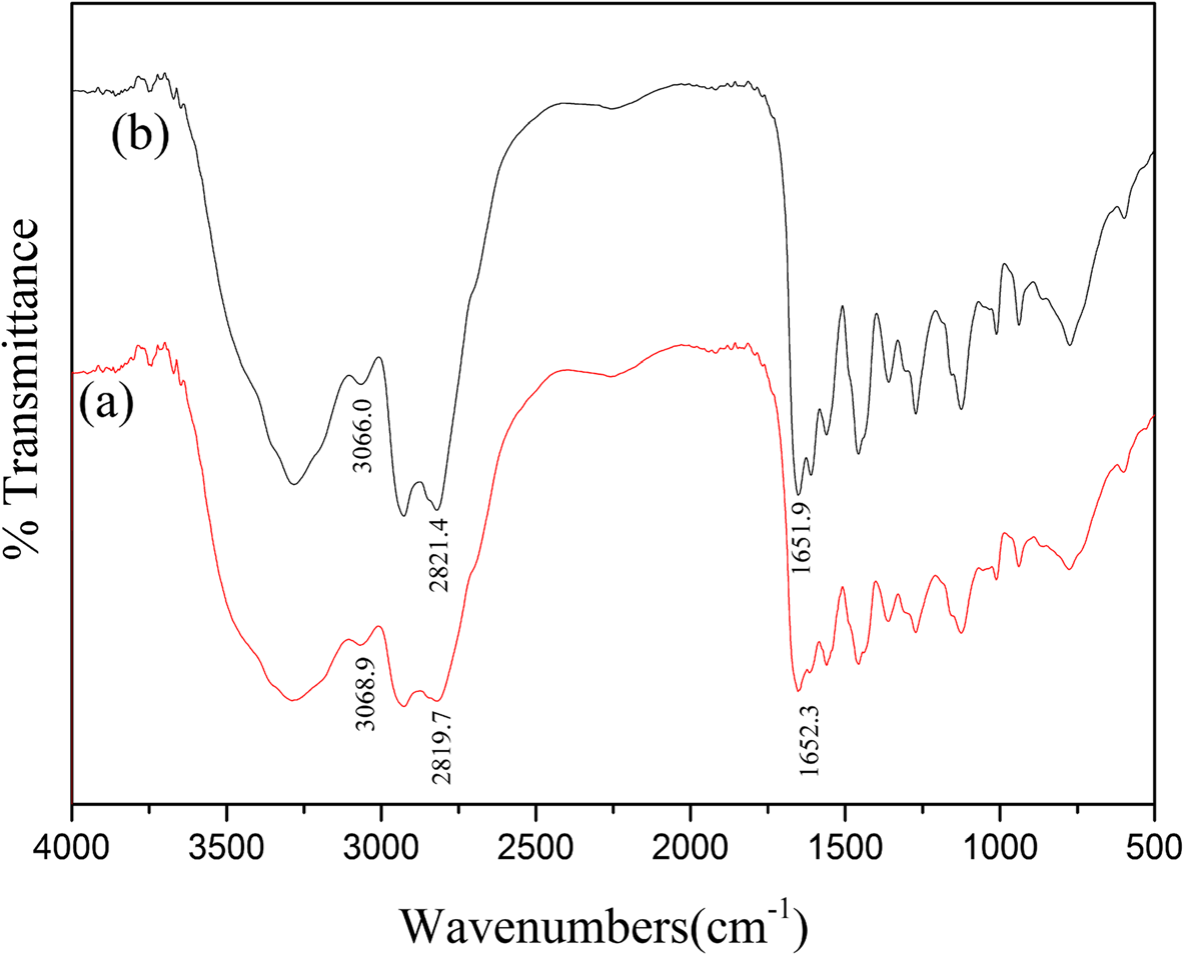
**FTIR spectra of (a) RSD-NH**_**2 **_**and (b) silver/RSD-NH**_**2 **_**nanohybrid.**

**Figure 3:**
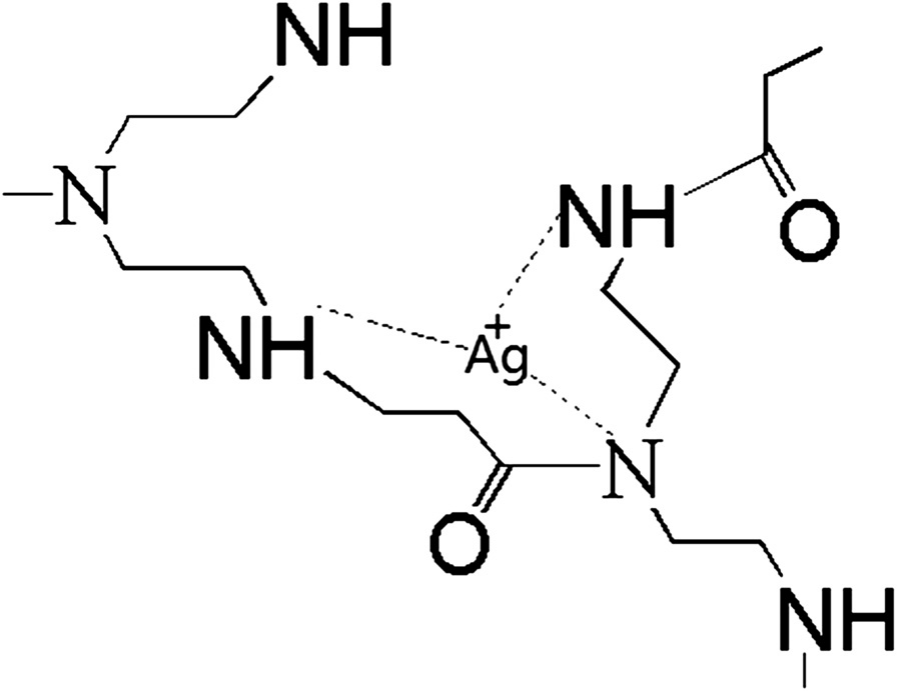
Schematic description of silver ammonia.

Figure 2 shows the FTIR spectra of RSD-NH_2_ and the resulting silver colloid. Comparing the spectra of the pure polymer and the silver/RSD-NH_2_ nanohybrid, the band positions of RSD-NH_2_ show an apparent shift. The band position at 3,068.9 cm^−1^, corresponding to amide B (NH stretching vibration modes) of RSD-NH_2_, shifted to a lower region (3,066 cm^−1^) after the formation of silver nanoparticles. The band position of CH_2_ symmetric stretching at 2,819.7 cm^−1^ shifted to 2,821.4 cm^−1^. The band position of amide I of RSD-NH_2_ at 1,652.3 cm^−1^ moved to a lower region (1,651.9 cm^−1^). It indicated that there are some interactions between the silver nanoparticles and RSD-NH_2_. The principle is illustrated in Figure 3: the molecule of RSD-NH_2_ contains numerous secondary and tertiary amine groups, as well as some primary amine groups at the peripheral region. These amine groups are able to attract silver ions and provide an electron source for the reduction process. After reduction, the silver nanoparticles can be formed in the cages by the amine groups, which can achieve functionalization for the nanohybrids. As the silver nanoparticles are confined in the interior of the polymers, their growth will be physically restricted by the meshes, so the size and size distribution can be effectively controlled.

**Figure 4:**
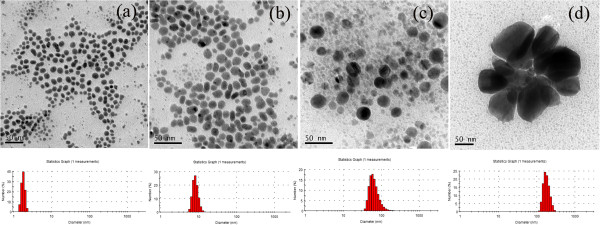
**TEM images and corresponding histograms of silver colloid nanoparticles [AgNO**_**3**_**] = 0.017 g/l (a), 0.085 g/l (b), 0.17 g/l (c), 0.225 g/l (d).**

Figure 4 shows the TEM images and the corresponding histograms of four samples prepared with four different initial AgNO_3_ concentrations. Upon increasing the initial AgNO_3_ concentrations from 0.017 to 0.17 g/l, the mean particle sizes increased from 1.76 to 65.77 nm, meanwhile the size distribution also increased. When the AgNO_3_ concentration is 0.225 g/l, some silver nanoparticles are more than 100 nm. The mean size of silver nanoparticles determined by DLS is consistent with the results by TEM images.

**Figure 5:**
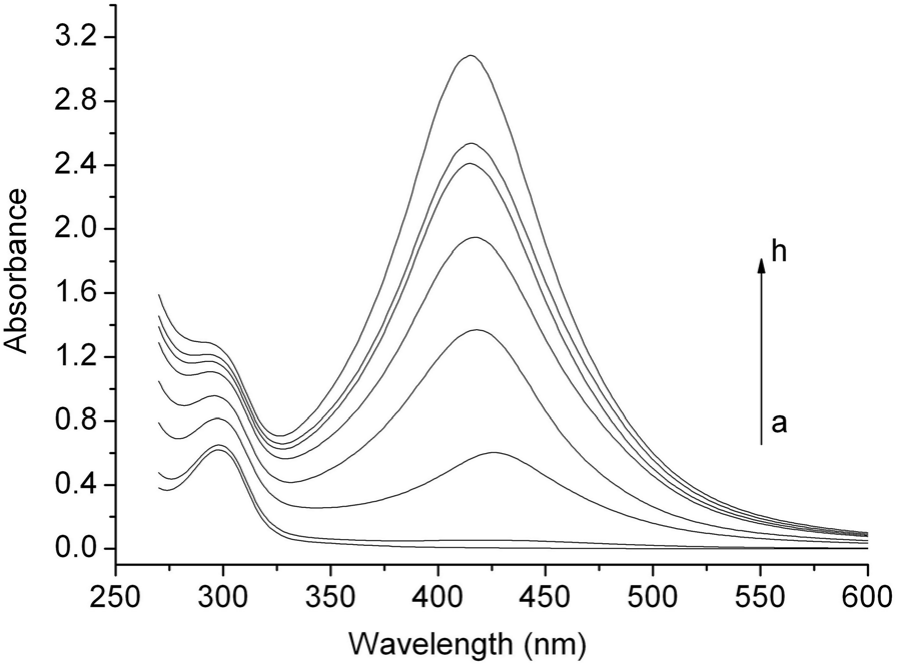
**UV-vis spectra of silver colloid nanoparticles at different time points. (a)** 0 h. **(b)** 1 h. **(c)** 6 h. **(d)** 12 h. **(e)** 24 h. **(f)** 48 h. **(g)** 1 week. **(h)** 2 weeks. [AgNO_3_] = 0.17 g/l.

Figure 5 shows the UV-vis spectra of silver nanoparticles recorded at different times during the preparation. At the beginning time, one characteristic peak at 298 nm is observed due to pure RSD-NH_2_[1]. As the stirring time increases, a new peak appears between 400 and 450 nm. This confirms the appearance of nanocrystallites of the silver particles in the solution; the shifting of peak positions with time also indicates the growing size of silver nanoparticles. Furthermore, the height of the absorption peaks of the silver nanoparticles increases and the full width at half maximum (FWHM) of the peaks decreases with time, which indicate the increasing amount and improved crystallinity of silver nanoparticles [16,17].

**Figure 6:**
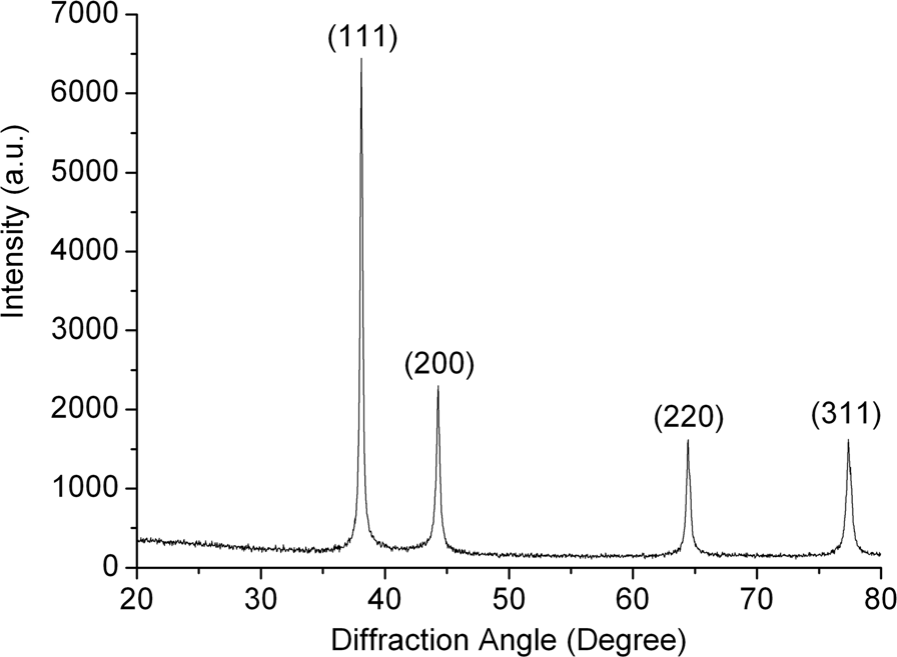
XRD spectra of silver nanoparticles.

The information given by TEM micrographs and UV-vis spectra indicates that the silver nanoparticles can be successfully synthesized through the reaction between AgNO_3_ and RSD-NH_2_. However, when the silver nanoparticle solution was non-intrusively placed for more than 24 h, a shining silver mirror appeared on the inner wall of the glass container and the color of the solution changed to black. This is due to the apparent agglomeration and oxidation of silver nanoparticles in the solution. We prepared silver nanoparticles with 0.085 g/l AgNO_3_, and the precipitated silver powders in the silver colloid were centrifuged, washed with methanol, and dried in air for XRD measurement. The result is shown in Figure 6. It clearly shows the (111), (200), (220), and (311) planes of the silver nanoparticles. As shown in Table 1, the size of silver nanoparticles calculated by using Scherrer's equation resulted in an average particle size of 26 nm. The mean size of silver nanoparticles calculated by Scherrer's equation is consistent with the results by TEM images. In a previous study, silk fabrics were immersed into a silver nanoparticle solution [18]. It turned out that the nanoparticles were aggregated and unevenly distributed on the surface of the fiber matrix. In this case, the silver nanoparticles may have loosely absorbed on the surface of fibers, making it difficult to continue the washing of fabrics. Therefore, we attempted the *in situ* synthesis of metal nanoparticles to reduce the metal ions directly on the matrix, which may form stronger binding between nanoparticles and fibers [19].

**Table 1:** Size of the micro-crystal of the resulting nanosilver particles

	**2*θ *(deg)**			
Planes	111	200	220	311
Half bandwidth	0.30	0.45	0.54	0.66
Size of the micro-crystal (nm)	26.74	17.66	20.96	21.71

### Characterization and antibacterial ability of in situ synthesized silver nanoparticles on silk fabrics

After the *in situ* reaction on the surface of silk fabrics was completed, the dried fabrics visually showed a bright yellow color. Generally, nanosilver particles are considered as a good antimicrobial agent on silk fabrics. To study the antimicrobial activities of silver nanoparticle-treated silk fabrics, *E. coli* and *S. aureus* were selected to perform antibacterial experiments.

Table 2 lists the whiteness index (WI), weight increase, and inhibition rates against *E. coli* and *S. aureus*, which were measured from the silver nanoparticle-treated silk fabrics by using 0.4 g/l RSD-NH_2_ solution with 0.0034, 0.0105, 0.017, 0.034, and 0.068 g/l AgNO_3_ solution. The samples are denoted accordingly as a, b, c, d, and e. As a reference, the whiteness of the original silk fabric is 90.79. As we can see in Table 2, the finished silk fabrics have excellent antibacterial rates against *E. coli* and *S. aureus*, which are more than 99%. When the silver content of silk fabrics was increased from 98.65 to 148.68 mg/kg, the antibacterial rate had no significant change, but the WI changed a little. Therefore, the silver nanoparticle-treated silk fabrics showed an excellent antibacterial property and satisfied whiteness when the AgNO_3_ concentration of the solution was low as shown in Table 2.

**Table 2:** The WI, silver content, and antibacterial rate of nanosilver-treated fabrics

**Samples**	**Silver content (mg/kg fabric)**	**WI**	**Antibacterial activities**			
			***S. aureus***		***E. coli***	
			**Surviving cells (CFU/ml)**	**% reduction**	**Surviving cells (CFU/ml)**	**% reduction**
Untreated	-	90.79	2.28 × 10^6^	-	4.37 × 10^6^	-
a	98.65	86.32	1.53 × 10^2^	99.99	2.22 × 10^3^	99.49
b	113.50	85.67	4.56 × 10^2^	99.98	2.09 × 10^3^	99.52
c	126.48	84.96	3.19 × 10^3^	99.86	1.39 × 10^3^	99.68
d	139.82	83.18	4.52 × 10^2^	99.98	9.1 × 10^2^	99.79
e	148.68	82.19	1.62 × 10^2^	99.99	8.7 × 10^2^	99.98

One of the most important features of nanosilver-treated silk fabrics is their durability against repeated washings. To study the washing durability, the nanosilver-treated silk fabrics were laundered 0, 5, 10, 20, and 50 times with detergents (Table 3). The silver content of 98.65 mg/kg on the finished silk fabric was selected in conducting the washing test. With the washing times increased, the silver content slightly decreased from 98.65 to 81.65 mg/kg while the corresponding whiteness increased. It is surprising that the antibacterial rate is still more than 97.43% for *S. aureus* and 99.86% for *E. coli* after 50 washings.

**Table 3:** The WI, silver content, and antibacterial rate of different washing times

**Silk samples**	**Laundering cycles**	**Silver content (mg/kg)**	**WI**	**Antibacterial activities**			
				***S. aureus***		***E. coli***	
				**Surviving cells (CFU/ml)**	**% reduction**	**Surviving cells (CFU/ml)**	**% reduction**
Untreated	-	-	90.79	2.28 × 10^6^	-	4.37 × 10^6^	-
Silver-treated fabrics	-	98.65	86.32	1.16 × 10^3^	99.49	8.74 × 10^2^	99.98
	5	95.02	86.43	3.44 × 10^3^	98.49	1.74 × 10^3^	99.96
	10	88.85	87.13	1.28 × 10^3^	99.49	6.11 × 10^3^	99.86
	20	87.14	87.58	2.53 × 10^3^	98.89	1.48 × 10^3^	99.96
	50	81.65	87.71	5.86 × 10^3^	97.43	6.11 × 10^3^	99.86

The excellent laundering durability of the silver nanoparticle-treated silk fabrics may be caused by the following reasons. Firstly, some imino groups of RSD-NH_2_ form a silver ammonia complex with silver nanoparticles, which easily penetrate into the amorphous zone of silk fibers. Secondly, silk is a protein fiber and amino acid is its basic structural unit, which has a large number of amino and carboxyl groups on the surface. The van der Waals force between molecules, as well as the hydrogen bond, will enhance the bonding between silver particles and silk fabrics [20].

**Figure 7:**
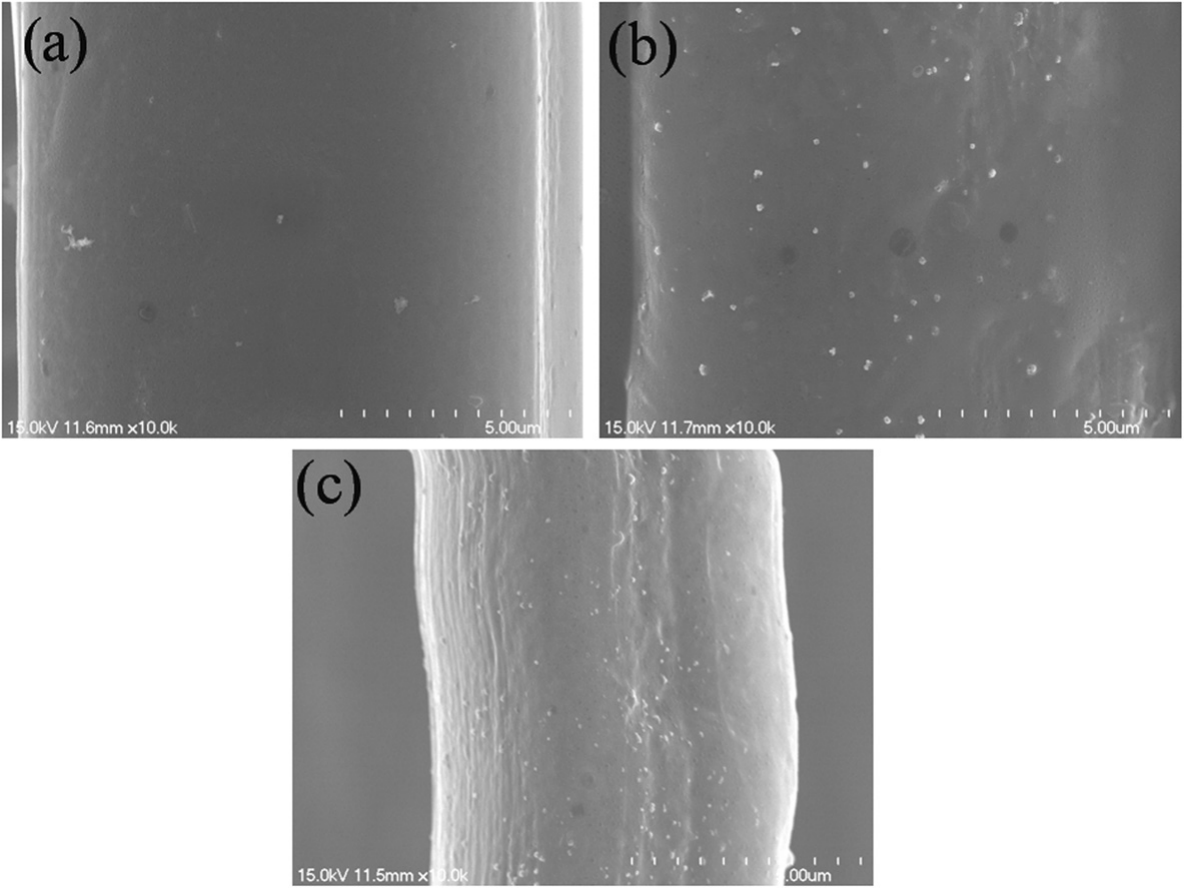
**SEM images of the surface of the silk fabrics. (a)** Original silk fabric. **(b)** Nanosilver-treated silk fabric. **(c)** Nanosilver-treated silk fabric after washing for 50 times.

**Figure 8:**
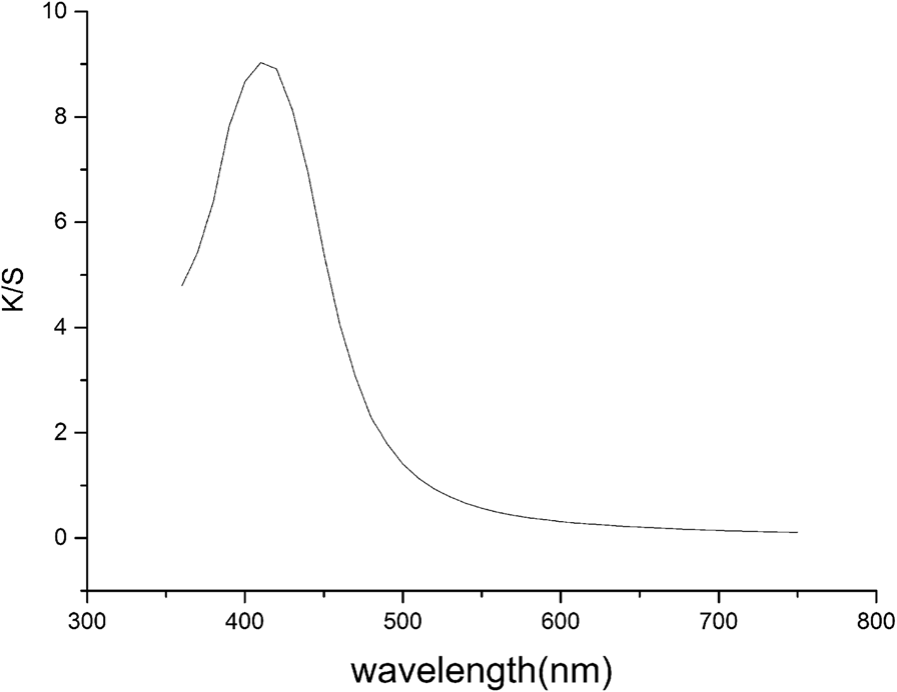
K/S spectrum of silver nanoparticle-treated silk fabrics.

The surface morphology of the original silk fabric and the silver nanoparticle-treated silk fabrics is compared in Figure 7. The synthesis condition of the silver nanoparticles is the mixture of 50 mg/l AgNO_3_ and 2 g/l RSD-NH_2_ solution. The scanning electron microscope images showed that silver nanoparticles distributed evenly on the surface of the silver nanoparticle-treated fabric. As the silver nanoparticle-treated silk fabric has good washing properties, silver nanoparticles can be found on the surface of the treated fabric even after washing for 50 times. Also, the K/S value indicates the presence of silver on the silk fabric. As shown in Figure 8, the obvious absorption peaks between 400- and 420-nm wavelength appeared in curves, which is consistent with the absorption peak of the silver nanoparticle solution [21]. Thus, we can deduce that there are indeed nanosilver particles on the surface of the silver-treated silk fabrics.

## Conclusions

A silver nanoparticle solution was prepared in one step by mixing AgNO_3_ and RSD-NH_2_ solution under vigorous stirring at room temperature. The multi-amino compound (RSD-NH_2_), which has abundant amino and imino groups, was synthesized by methacrylate and polyethylene polyamine in methanol. The formation of silver nanoparticles was characterized by various methods. However, the results indicated that silver nanoparticles easily agglomerate in ambient condition. Therefore, an *in situ* synthesis method was conducted through the reaction between the multi-amino compound (RSD-NH_2_) and the silver nitrate solution. The surface morphology, whiteness, silver content, antibacterial activity, and washing durability of nanosilver-treated fabrics were examined. The experimental results confirmed that the *in situ* synthesized silver nanoparticles evenly distributed on the surface of fibers. The inhibition zone and the antibacterial rate demonstrated that the finished fabrics have an excellent antibacterial property against *S. aureus* and *E. coli*. When the nanosilver-treated fabric which included a silver content of 98.65 mg/kg was washed 50 times, the silver content slightly decreased from 98.65 to 81.65 mg/kg and the corresponding whiteness increased. However, it is surprising that the antibacterial rate is still more than 97.43% for *S. aureus* and 99.86% for *E. coli* after 50 washings.

## Competing interests

The authors declare that they have no competing interests.

## Authors’ contributions

GZ and YL carried out the experiments and measurements and drafted the manuscript. XG participated in the discussion. YC contributed to the design of the experiment and analysis of the results in this paper. All authors read and approved the final manuscript.
